# Thoracoscopic wedge resection of the upper lobe of the left lung achieved by a new approach of laryngeal mask general anesthesia with preservation of spontaneous breathing: A case report

**DOI:** 10.1097/MD.0000000000039172

**Published:** 2024-08-02

**Authors:** Mengran Xie, Yu Meng, Huanshuang Pei

**Affiliations:** a Department of Anesthesiology, The Fourth Hospital of Hebei Medical University, Shijiazhuang, China.

**Keywords:** laryngeal mask airway, preservation of spontaneous respiration, remimazolam, surface anesthesia of the pharynx, thoracic paravertebral nerve block

## Abstract

**Introduction:**

General laryngeal mask anesthesia with the preservation of spontaneous breathing has accelerated the advancement of the enhanced recovery after surgery concept in thoracoscopic surgery. However, the need for increased doses of anesthetic drugs to reduce laryngeal mask airway (LMA) stimulation poses challenges due to the increased risk of hypotension, respiratory depression, susceptibility to hypoxemia, and carbon dioxide retention, particularly in the lateral position.

**Patient concerns:**

During the perioperative period, reducing the dose of anesthetic drugs while simultaneously improving LMA tolerance and preventing circulatory and respiratory depression poses a challenge.

**Diagnoses:**

The patient was diagnosed with a nodule in the upper lobe of the left lung.

**Interventions:**

In this case, we chose remimazolam sedation, which mildly inhibits circulatory respiration, and used mucosal surface anesthesia in the pharynx. This approach improved the patient’s tolerance to LMA, reduced the dose of anesthetic drugs, and facilitated the successful thoracoscopic wedge resection of the upper lobe of the left lung with preservation of spontaneous respiration.

**Outcomes:**

During 2 weeks follow-up, the patient recovered satisfactorily and did not report any discomfort.

**Conclusion:**

We used pharyngeal mucosal surface anesthesia and thoracic paravertebral nerve block in combination with remimazolam sedation to provide precise analgesia, moderate sedation, and successful LMA general anesthesia with preservation of spontaneous respiration in patients undergoing thoracoscopic pulmonary wedge resection.

## 1. Introduction

The advancement of the concept of enhanced recovery after surgery (ERAS) has propelled the adoption of general anesthesia using a laryngeal mask with the preservation of spontaneous respiration both domestically and internationally. This approach offers low stimulation, high comfort, and the ability to avoid airway damage associated with 1-lung ventilation. However, to reduce laryngeal mask airway (LMA) stimulation, the dose of anesthetic drugs should be increased, maintaining the clinical use of propofol, remifentanil, or inhalation anesthesia. Nevertheless, increasing the dosage of anesthetic drugs increases the risk of hypotension and respiratory depression, especially in the lateral position, which is more prone to hypoxemia and carbon dioxide retention. Therefore, the challenge lies in reducing the dose of anesthetic drugs while simultaneously improving the tolerance of LMA to prevent circulatory and respiratory depressions. Remimazolam is a new ultrashort-acting benzodiazepine with a rapid onset of action, short half-life, minimal effect on respiratory circulation, low accumulation over long infusion periods, and antagonist activity. Literature suggests that combining pharyngeal surface anesthesia with remimazolam sedation results in smoother hemodynamics during induction.^[1]^ In this case, we propose a new approach to general anesthesia using a laryngeal mask with preservation of spontaneous respiration, pharyngeal mucosal surface anesthesia, and thoracic paravertebral nerve block (TPVB) combined with remimazolam sedation. This approach aims to provide precise analgesia, moderate sedation, and successful LMA general anesthesia to preserve spontaneous respiration in patients undergoing thoracoscopic pulmonary wedge resection.

## 2. Case presentation

A 54-year-old man (height: 166 cm; weight: 70 kg) was admitted to our hospital with a 6-month-old pulmonary nodule detected in the upper lobe of his left lung, which was diagnosed during a physical examination. He presented with no cough, sputum, chest tightness, shortness of breath, fatigue, night sweats, low-grade fever in the afternoon, chest or back pain, hoarseness, and difficulty in drinking water. Chest computed tomography revealed the following findings: grinding glass density nodules in the apical posterior segment of the left upper lobe of the lung, suggestive of glandular precursor lesions; multiple tiny nodules in the left lung; and a few striped shadows in the middle lobe of the right lung and the lingual segment of left lung upper lobe. The patient was admitted to our hospital as an outpatient for further treatment of the “nodules in the upper lobe of the left lung.” The patient had a 6-month history of type 2 diabetes mellitus, with a maximum fasting blood glucose level of 11 mmol/L. The patient was prescribed metformin and acarbose orally on weekdays, and he reported satisfactory blood glucose control. The patient had no history of hypertension or coronary heart disease. During physical examination, his temperature was 36.6°C, pulse rate was 82 beats/min, respiratory rate (RR) was 20 breaths/min, and blood pressure (BP) was 115/85 mm Hg. Cardiopulmonary examination revealed no obvious abnormalities. Pulmonary function testing revealed that ventilatory function was normal, small airway function was normal, lung residual air volume was not increased, total diffusion function was not reduced, and unit alveolar diffusion function was intact, a forced expiratory volume in 1 second: 3.30 L, a forced expiratory volume in 1 second/forced vital capacity of 83.31%. Electrocardiography showed sinus rhythm with normal electrocardiography. Cardiac assessment revealed a small amount of mitral regurgitation and mildly reduced left heart diastolic function, a left ventricular ejection fraction of 64%. Blood routine showed hemoglobin level of 143.0 g/L, hematocrit (HCT) level of 42.8%. Color ultrasonography of the deep veins of both lower limbs revealed widening of the left calf muscle veins and stagnant blood flow. The preliminary diagnosis was a nodule in the left upper lung lobe. Thoracoscopic wedge resection of the upper lobe of the left lung, with preservation of spontaneous respiration under general anesthesia under hook-and-loop positioning, was planned.

**Figure 1.**
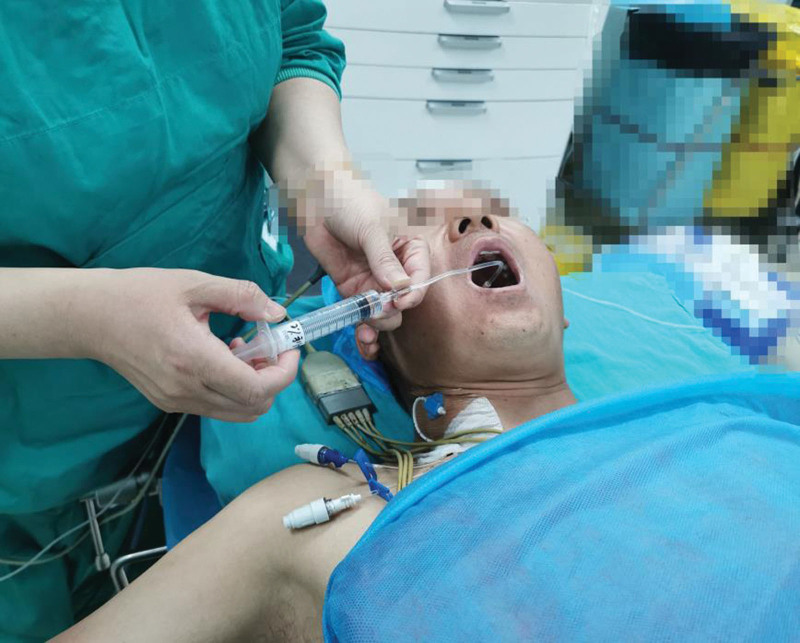
Pharyngeal mucosal surface anesthesia.

Upon admission, the patient’s left upper extremity venous access was established. Vital signs were recorded as follows: heart rate (HR) 68 beats/min, BP 138/91 mm Hg, SpO_2_ 98%, and bispectral index (BIS) 98. The left radial artery was punctured and cannulated to monitor the invasive arterial BP (ABP), and the arterial blood gases (AGB) were measured, revealing FiO_2_ of 0.21, PaO_2_ of 71.6 mm Hg, PaCO_2_ of 37.2 mm Hg, Glu of 6.27 mmol/L and HCT level of 41.5%. The patient was instructed to take 5 mL of dacronin gelatin paste and swallow after 10 minutes. Mucosal surface anesthesia in the pharynx was achieved with the administration of 5 mL of 2% lidocaine via laryngeal anesthesia tube (Fig. 1). After 5 minutes, the patient reported numbness in the pharynx without respiratory distress. The patient was instructed to cough and expectorate to clear the respiratory secretions, and then 1 mg of pentylenetetrazol hydrochloride was injected to dry the airway. Before the induction of anesthesia, 70 mg of flurbiprofen axetil was administered for 1 minute. The patient was instructed to lie on the right side, and ultrasound-guided left T_6_ TPVB with 0.185% bupivacaine 20 mL was performed. Subsequently, the patient was instructed to lie flat. The thoracic paravertebral anesthesia planes were tested 15 minutes after the nerve block, and the range of the temperature sensing planes was up to T_3_-T_9_, which provided a perfect regional block for the surgery.

**Figure 2.**
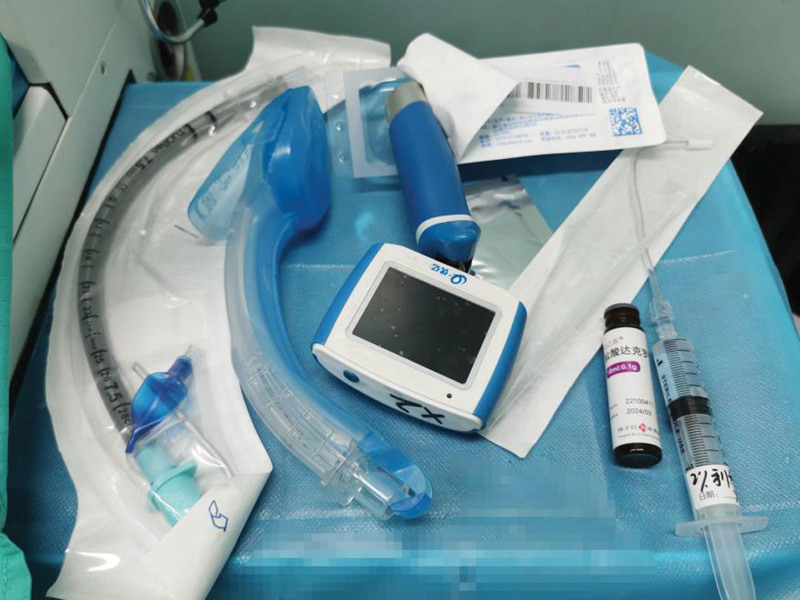
Preparation of airway tools.

Anesthesia was induced as follows: a static injection of remimazolam 15 mg was administered, and the patient received oxygen and denitrogenation via mask for 2 minutes until the BIS reached 40. Subsequently, an LMA 4^#^ (Tianjin Medical Corp.) (Fig. 2) was inserted orally, allowing the patient to maintain spontaneous respiration. The patient experienced slight movement after the insertion of LMA 4^#^, which disappeared after the injection of 50 mg of propofol. The respiratory circuit was then connected and fixed with an FiO_2_ of 1.0, an oxygen flow rate of 1.5 L/min, an adjustable pressure limiting valve set to 0 mm Hg, VT 350 to 400 mL, and an RR of 12 to 16 breaths/min. After deepening the anesthesia with an additional administration of 50 mg of propofol, the patient was positioned on his right lateral side. His vital signs were stable, with no body movements. Anesthesia was maintained using a remimazolam pump at a rate of 0 to 2 mg.kg^−1^-h^−1^ to maintain SpO_2_ >93% and BIS 45 to 60. The pumping rate of remimazolam was adjusted according to BIS readings and vital signs, and respiration was manually assisted if respiratory depression occurred. An incision of approximately 2 cm in length was made in the 4th intercostal space in the anterior axillary line as an operating hole. Upon entering the chest cavity, the surgeon applied 5 mL of 2% lidocaine on the lung surface to provide surface anesthesia without vagal blockade. Throughout the lung wedge resection procedure, the patient’s respiratory amplitude was slightly elevated, with ABP at 125/70 mm Hg, HR at 75 beats/min, SpO_2_ at 96%, RR at 15 breaths/min, and BIS at 55. Considering that this was not related to the vagus nerve blockade, the patient was injected with remifentanil at a rate of 0.05 to 0.20 μg.kg^−1^.min^−1^, which resulted in significant improvement. The anesthesia procedure proceeded smoothly, with intraoperative fluctuations in ABP and HR remaining <20% of the baseline values while SpO_2_ was at 94% to 99% and RR was at 12 to 15 breaths/min, BIS was maintained at 45 to 60, with no incidence of choking of body movement. AGB analysis conducted 5 minutes into the surgery revealed FiO_2_ of 0.3, PaO_2_ of 196.8 mm Hg, PaCO_2_ of 46.5 mm Hg, Glu of 5.84 mmol/L, and HCT level of 38.29%. Subsequently, AGB analysis at 30 minutes after surgery showed FiO_2_ of 0.3, PaO_2_ of 72.85 mm Hg, PaCO_2_ of 64.1 mm Hg, Glu of 6.35 mmol/L, and HCT level of 38.99%. Five minutes before the end of the surgery, the pumping of remazolam was halted, and the lungs were expanded for 30 seconds, with an airway pressure of 25 mm Hg. Following the surgery, the patient resumed the supine position, and received an injection of flumazenil (0.5 mg). After 1 minute, the patient’s eyes opened, and he was able to nod his head in response to instructions. Additionally, his spontaneous respiration was good, with an HR of 101 beats/min, ABP measuring 131/71 mm Hg, SpO_2_ at 95%, RR at 14 beats/min, and BIS at 94. After removing the LMA, the patient was observed for 15 minutes, during which the visual analog scale pain score was 2, HR was 85 beats/min, ABP was 142/81 mm Hg, SpO_2_ was 99%, and BIS was 98. The patient was asked to report the disappearance of throat numbness and to indicate if he experienced any discomfort, such as sore throat or hoarseness. A modified Aldrete score of ≥9 was recorded. The patient did not experience any further sedation, respiratory and/or circulatory depression during the course of observation.

After the removal of the radial artery tube, pressure was applied to control bleeding, and the patient was transferred back to the ward. Upon returning to the room, the patient’s vital signs remained stable, and there was no report of discomfort. The surgical procedure lasted 50 minutes, and the anesthesia lasted 110 minutes with remimazolam dosage at 65 mg, propofol dosage at 100 mg, remifentanil dosage at 100 μg, and flurbiprofen axetil at 70 mg. The intraoperative fluid infusion was 900 mL, with a recorded blood loss of 10 mL. At the end of the surgery, the patient was administered intravenous self-controlled analgesia.

Chest radiography conducted on the day following the surgery revealed postoperative changes in the left lung and a few striated shadows in the middle lobe of the right lung, similar to the preoperative findings. The patient recovered satisfactorily and was discharged on the second day after the surgery. At the 2-week postoperative follow-up, the patient had recovered satisfactorily, without any pulmonary complications (Table 1).

**Table 1:** Case report timeline.

Item		Timeline
Preoperative	1	The patient was admitted to the hospital with a 6-mo-old pulmonary nodule in the upper lobe of his left lung.
	2	The patient had a history of type 2 diabetes mellitus and reported good glycemic control with no history of hypertension or coronary heart disease.
	3	Chest computed tomography revealed grinding glass density nodules in the apical posterior segment of the left upper lobe of the lung.
	4	Thoracoscopic wedge resection of the upper lobe of the left lung with preservation of spontaneous respiration under general anesthesia under hook-and-loop positioning was planned.
Perioperative	5	After admission, the patients’ left upper extremity venous access was opened and BP, ECG, SpO_2_, and BIS were routinely monitored.
	6	Invasive blood pressure was monitored and arterial blood gas analysis was conducted.
	7	Pharyngeal mucosal surface anesthesia, ultrasound-guided TPVB, and subsequent induction of anesthesia with sedation of remimazolam were performed sequentially.
	8	Airway tool of choice: LMA4^#^
	9	After insertion of the laryngeal mask and before positioning, 50 mg of propofol was added to deepen anesthesia.
	10	When a further lobectomy wedge was performed, the patient’s respiratory amplitude was slightly greater and improved with the pumping of an appropriate amount of remifentanil.
	11	In this case, general anesthesia using pharyngeal surface anesthesia, TPVB in combination with remimazolam sedation LMA provided definitive analgesia, moderate sedation, and successful anesthesia with preservation of spontaneous respiration for thoracoscopic lung wedge surgery. The patient’s intraoperative vital signs were stable, with no respiratory depression.
Postoperative	12	At the end of the surgical procedure, The patient was injected with flumazenil 0.5 mg. After 1 min, the patient’s eyes were opened, and he could nod his head in response to instruction, The patient recovered successfully with no respiratory complications.
	13	The patient was discharged on the second day after surgery.
	14	No pulmonary complication was observed during the 2-wk postoperative follow-up.

BIS = bispectral index, LMA = laryngeal mask airway, TPVB = thoracic paravertebral nerve block.

## 3. Discussion

With the extension of the ERAS concept, traditional open thoracic surgery has gradually been replaced by minimally invasive thoracoscopic surgery and has evolved from multi-hole to single-hole lumpectomy. This shift significantly reduces surgical trauma and shortens hospitalization duration.^[2]^ Preservation of spontaneous breathing under nonintubated general anesthesia involves the insertion of an LMA while the patient is under anesthesia. This ensures the maintenance of spontaneous breathing intraoperatively and mitigates the risk of airway damage associated with 1-lung ventilation.^[3]^ Studies have shown that preserved spontaneous breathing with nonintubated anesthesia, as part of the concept of ERAS, is both safe and feasible for thoracoscopic lung cancer surgery. It offers advantages over intubated anesthesia, including reduced healthcare costs.^[4]^

Total intravenous anesthesia or general anesthesia with LMA ventilation combined with TPVB is the primary modality of general anesthesia, with the preservation of spontaneous breathing. However, the current protocols of fewer opioids combined with propofol or static aspiration combined anesthesia have different degrees of respiratory depression, which increases the risk of intraoperative hypoxemia and hypercapnia. Simultaneously, respiratory depression caused by anesthetic drugs and surgical compression of the lung tissues increases the risk of postoperative lung expansion difficulties and postoperative pulmonary atelectasis.

Providing definitive analgesia without compromising respiration function is crucial for thoracoscopic surgery, along with the preservation of spontaneous breathing. Commonly used opioids can lead to adverse effects such as constipation, nausea and vomiting, drowsiness, nociceptive sensitization, and pruritus.^[5]^ Escitalopram poses the disadvantage of inducing a dissociated anesthetic state and triggering adverse emotional reactions after anesthesia. The combination of dexmedetomidine with nerve block increases the risk of dry mouth, hypotension, and bradycardia in patients.^[6]^ In this case, ultrasound-guided TPVB was performed while the patient was awake, facilitating precise visualization and block-plane testing to confirm accuracy. Simultaneously, it does not compromise respiratory circulation, thus increasing the intraoperative safety and postoperative comfort of the patient. Preoperative administration of the nonsteroidal analgesic drug flurbiprofen axetil has hyperalgesia as well as an improved oxygenation effect, which can improve the quality of anesthesia in patients. It exhibits potent analgesic and sedative effects, reduces the inflammatory response, has good lung protection effect, and offers enhanced safety.^[7]^

Moderate sedation is essential to ensure the successful completion of the procedure. Research has shown that increasing the dose of anesthetics during induction reduces somatic movements during LMA placement; it also increases the risk of respiratory depression and lowers BP. Remimazolam is a new type of ultrashort-acting benzodiazepine with rapid onset of action, short half-life, low impact on the respiratory cycle, and low accumulation over long infusions and antagonists.^[8]^ The sedative effects can be rapidly reversed by flumazenil.^[9]^ Advising patients to use a precontained dacronine gelatin paste and administering intravenous remimazolam and a 5 mL topical spray of 2% lidocaine in the oropharynx prior to induction resulted in smoother hemodynamics during induction.^[1]^ In this case, we chose remimazolam sedation with mild respiratory inhibition. We used full epineural anesthesia to enhance the patient’s tolerance to LMA placement, reduced the dosage of remimazolam, and avoided excessively deep anesthesia to prevent aggravating the inhibition of voluntary respiration. Consequently, we successfully preserved voluntary anesthesia with no occurrence of hypoxemia, carbon dioxide accumulation, apnea, or the need for hand-control-assisted respiration. This was achieved alongside definitive analgesia provided through thoracic pars interspinalis blockade. Respiratory anesthesia was successfully achieved. However, this protocol has been rarely reported. Intraoperative adjustment of the remimazolam pumping rate maintained the depth of sedation at 45 to 60, which allowed for simultaneous elimination of both episodic and implicit memory^[10]^ and without excessive respiratory depression.^[11]^ During sedation with remimazolam, it is necessary to remedy the shallow anesthesia. Key steps include inserting the LMA in the prone position, positioning the patient, and making an incision into the thorax. If shallow anesthesia occurs, an appropriate amount of propofol can be added.

With the preservation of spontaneous breathing under anesthesia, the patient’s spontaneous breathing excessive respiratory effort can pose challenges during thoracic surgery. Common methods to mitigate this situation include performing a thoracoscopic vagal block (for lobectomy or hilar operation), spraying lidocaine on the lung surface (for alveolus or lung wedge resection), and administering opioids. In this case of lung wedge resection, the surgeon sprayed lidocaine on the lung surface without performing vagal block. During the surgical procedure, respiratory amplitude increased slightly during the lung wedge resection. Subsequently, remifentanil was administered at a rate of 0.05 to 0.20 μg.kg^−1^.min^−1^, resulting in significant improvement.

Preservation of autonomous breathing with LMA anesthesia is highly susceptible to hypercapnia in thoracic surgery; mild-to-moderate hypercapnia is permissible in thoracic surgery and is one of the strategies for lung-protective ventilation. Other studies have indicated that elevated PaCO_2_ increases volumes_middle cerebral arter_ (V_MCA_), thereby improving cerebrovascularreactivity-CO_2_ (CVR-CO_2_), significantly enhancing mini-mental state examination scores, and reducing the incidence of early postoperative cognitive dysfunction on postoperative days 1 and 7.^[12]^ AGB analysis at different times during surgery in this case showed that PaCO_2_ was within the permissible range, and CO_2_ returned to normal after the resumption of 2-lung ventilation.

Maintaining autonomous breathing during anesthesia presents challenges, particularly in managing secretion retention, which may lead to increased intraoperative airway pressure, impede lung expansion, and increase the risk of postoperative pulmonary atelectasis. In this case, the preoperative focus was on clearing respiratory secretions. Before induction, the patient was instructed to cough and expectorate and receive Changtorin sedation. Throughout the procedure, BIS was monitored to ensure that the depth of anesthesia was precise, and postoperatively, the patient was again instructed to cough and expectorate after waking up at the end of the surgical procedure, which greatly reduced the risk of secretion retention.

In this case, the patient’s tolerance to LMA was increased by administering adequate pharyngeal epineurial anesthesia. Intravenous injection of remimazolam was used to achieve moderate sedation, and propofol and remifentanil were used as remedial medication. A single anesthetic medication with a short-acting effect and flumazenil antagonist was administered at the end of the surgical procedure. The patient regained consciousness promptly without experiencing any agitation, sore throat, hoarseness, or postoperative nausea or vomiting. However, there was a slight transient increase in respiratory amplitude during the operation, possibly attributed to the absence of a vagus nerve block. Therefore, this aspect should be addressed in future clinical studies. While this case report provides clinical insight, further clinical trials are necessary to validate these findings. It should be noted that awake thoracoscopic wedge resection surgery is not a form of anesthesia management that can be applied to every patient. It should be kept in mind that the patient was a cardiac stable patient with good general condition and good pulmonary function tests. This procedure cannot be applied to every patient and this is a limitation of the study.

In conclusion, in this case, general anesthesia using pharyngeal surface anesthesia, TPVB in combination with remimazolam sedation, and LMA facilitated definitive analgesia, moderate sedation, and successful anesthesia with preservation of spontaneous respiration for thoracoscopic lung wedge surgery. In addition, pharyngeal mucosal surface anesthesia enhances the patient’s tolerance to LMA and reduces the need for remimazolam, thereby contributing to the preservation of spontaneous breathing, improving the patient's prognosis, and accelerating the course of ERAS.

## Author contributions

**Writing—original draft:** Mengran Xie, Huanshuang Pei.

**Writing—review & editing:** Mengran Xie, Huanshuang Pei.

**Investigation:** Yu Meng.
